# The Normal Limits, Subclinical Significance, Related Metabolic Derangements and Distinct Biological Effects of Body Site-Specific Adiposity in Relatively Healthy Population

**DOI:** 10.1371/journal.pone.0061997

**Published:** 2013-04-19

**Authors:** Chun-Ho Yun, Hiram G. Bezerra, Tung-Hsin Wu, Fei-Shih Yang, Chuan-Chuan Liu, Yih-Jer Wu, Jen-Yuan Kuo, Chung-Lieh Hung, Jason Jeun-Shenn Lee, Charles Jia-Yin Hou, Hung-I Yeh, Chris T. Longenecker, Ricardo C. Cury

**Affiliations:** 1 Department of Biomedical Imaging and Radiological Sciences, National Yang Ming University, Taipei, Taiwan; 2 Department of Radiology, Mackay Memorial Hospital, Taipei, Taiwan; 3 Department of Medicine, Mackay Medical College, and Mackay Medicine Nursing and Management College, Taipei, Taiwan; 4 Harrington Heart and Vascular Institute, University Hospitals Case Medical Center, Cleveland, Ohio, United States of America; 5 Graduate Institute of Health Care Organization Administration, College of Public Health National Taiwan University, Taipei, Taiwan; 6 Health Evaluation Center, Mackay Memorial Hospital, Taipei, Taiwan; 7 Department of Medical Technology, Yuanpei University of Science and Technology, Hsin-Chu, Taiwan; 8 Division of Cardiology, Department of Internal Medicine, Mackay Memorial Hospital, Taipei, Taiwan; 9 Department of Health Industry Management, Kainan University, Taoyuan, Taiwan; 10 Cardiovascular MRI and CT Program, Baptist Cardiac Vascular Institute, Miami, Florida, United States of America; Indiana University School of Medicine, United States of America

## Abstract

**Background:**

The accumulation of visceral adipose tissue that occurs with normal aging is associated with increased cardiovascular risks. However, the clinical significance, biological effects, and related cardiometabolic derangements of body-site specific adiposity in a relatively healthy population have not been well characterized.

**Materials and Methods:**

In this cross-sectional study, we consecutively enrolled 608 asymptomatic subjects (mean age: 47.3 years, 27% female) from 2050 subjects undergoing an annual health survey in Taiwan. We measured pericardial (PCF) and thoracic peri-aortic (TAT) adipose tissue volumes by 16-slice multi-detector computed tomography (MDCT) (Aquarius 3D Workstation, TeraRecon, San Mateo, CA, USA) and related these to clinical characteristics, body fat composition (Tanita 305 Corporation, Tokyo, Japan), coronary calcium score (CCS), serum insulin, high-sensitivity C-reactive protein (Hs-CRP) level and circulating leukocytes count. Metabolic risk was scored by Adult Treatment Panel III guidelines.

**Results:**

TAT, PCF, and total body fat composition all increased with aging and higher metabolic scores (all p<0.05). Only TAT, however, was associated with higher circulating leukocyte counts (ß-coef.:0.24, p<0.05), serum insulin (ß-coef.:0.17, p<0.05) and high sensitivity C-reactive protein (ß-coef.:0.24, p<0.05). These relationships persisted after adjustment in multivariable models (all p<0.05). A TAT volume of 8.29 ml yielded the largest area under the receiver operating characteristic curve (AUROC: 0.79, 95%CI: 0.74–0.83) to identify metabolic syndrome. TAT but not PCF correlated with higher coronary calcium score after adjustment for clinical variables (all p<0.05).

**Conclusion:**

In our study, we observe that age-related body-site specific accumulation of adipose tissue may have distinct biological effects. Compared to other adiposity measures, peri-aortic adiposity is more tightly associated with cardiometabolic risk profiles and subclinical atherosclerosis in a relatively healthy population.

## Introduction

Aging remains one of the strongest determinants of cardiovascular risk worldwide[1] Advancing age has been associated with endothelial dysfunction, atherosclerosis[2] and cumulative risk of coronary artery events in both men and women.[2] Thus, aging is an important component of global cardiovascular risk scores, including the Framingham risk score and the European SCORE, in asymptomatic patients.[3] The mechanism of increased cardiovascular risk with aging is incompletely understood, though recent studies have suggested that reduced baseline metabolic rates with aging may lead to subsequent metabolic abnormalities and obesity.[4]

There is growing evidence that excessive visceral adipose tissue (including visceral abdominal, pericardial, and thoracic peri-aortic adipose tissue) is linked to abnormal lipid profiles, enhanced systemic inflammation, diabetes and cardiovascular diseases.[5]–[7] A preferential increase in abdominal visceral adipose tissue (VAT) with normal aging is associated with a variety of metabolic complications in the elderly,[8] when compared with younger BMI-matched populations.[9]–[13] Furthermore, serum C-reactive protein, a well-recognized biomarker of systemic inflammation and unstable plaque, is elevated in both elderly genders with metabolic syndrome or central obesity.[14],[15]

Although accumulating evidence supports a link between excessive pericardial adipose tissue and higher prevalence of atherosclerosis[7], [16], recent data have suggested varied biological effects of different visceral adipose tissue depots due to their diverse physiologic roles.[17] For example, pericardial adipose tissue may have both cardioprotective and cardiotoxic effects, and discrepancies in the literature regarding the association of pericardial adipose tissue with cardiovascular disease may be due to differences in the patient population studied or different anatomic definitions and methodologies.[16], [18], [19] To date, the biologic effects of different visceral adipose tissue depots in a relatively healthy population have not been well-defined. Early recognition of abnormal visceral adipose tissue deposition, either in terms of total volume burden or anatomic preference, may aid in risk stratification and cardiovascular prevention efforts.

In this study, we therefore sought to define the relationships between visceral adipose tissue depots and measures of cardiometabolic risk in a relatively healthy population undergoing primary cardiovascular health surveillance.

## Materials and Methods

### Study population

The study was approved by the Institutional Review Board of Mackay Memorial Hospital, Taipei, Taiwan. All participants signed written informed consent prior to examinations. Data were analyzed anonymously. From 2005 to 2009, a total of 2050 consecutive subjects underwent cardiovascular health survey at our center that included a non-contrast enhanced computed tomography (CT) scan of the heart for coronary calcium scoring. Apparently healthy, normotensive, and non-obese (BMI<30 kg/m^2^) subjects were eligible for the current study. Additional exclusion criteria included use of lipid-lowering medication or any known history of diabetes, angina symptoms, or other cardiovascular diseases including myocardial infarction, coronary arterial disease, stroke, atrial fibrillation, congestive heart failure history, or peripheral arterial disease. Finally, subjects who had evidence of advanced subclinical coronary atherosclerosis on the CT scan (coronary calcium score≧100) were excluded from this study.

Baseline demographics and medical history were obtained along with a detailed physical exam. Structured questionnaires were used to quantify self-reported alcohol consumption, smoking and physical activity. A variety of anthropometric measures including height, weight, waist and hip circumferences were obtained. Resting blood pressures were measured by medical staff using a standardized sphygmomanometer. Metabolic risk/score was calculated as the total number of abnormal items of the National Cholesterol Education Program Adult Treatment Panel III criteria for metabolic syndrome[20]: (1) waist circumference ≥80 cm for women or ≥90 cm for men; (2) fasting glucose ≥100 mg/dL; (3) HDL <40 mg/dL for men or <50 mg/dL for women; (4) triglycerides >150 mg/dL; and/or (5) blood pressure >130/85 mmHg. The metabolic score therefore ranged from 0 to 5.[21] The presence of metabolic syndrome (MetS) was defined as a metabolic score of 3 or more.

### Biochemical data and serum markers of cardiometabolic risk

Venous blood sampling and analysis were performed according to the Clinical Laboratory Standards Institute guidelines (Specimen Choice, Collection, and Handling; Approved Guideline H18-A3). To ensure accuracy, results were verified by repeating the tests on the same tube one day later. A Hitachi 7170 Automatic Analyzer (Hitachi Corp. Hitachinaka Ibaraki, Japan) was used to measure fasting glucose (hexokinase method), post-prandial glucose, HbA1c, uric acid, blood urea nitrogen, creatinine (kinetic colorimetric assay), homocysteine, and lipid profile (homogeneous enzymatic colorimetric assay). High-sensitivity C-reactive protein (hs-CRP) was measured by latex particle-enhanced immunoassay (Elecsys 2010; Roche, Mannheim, Germany). Estimated glomerular filtration rate (eGFR) was calculated using the Modification of Diet in Renal Disease equation. HOMA-IR was calculated using the following formula: HOMA-IR  =  fasting glucose (mg/dl) × fasting insulin (µU/ml)/405).[22]

### Coronary Calcium Score (CCS) Measurement

Multi-detector CT (MDCT) of the heart was performed using a 16-slice scanner (Sensation 16, Siemens Medical Solutions, Forchheim, Germany) with 16×0.75 mm collimation, rotation time 420 ms and tube voltage of 120 kV. In one breath-hold, images were acquired from above the level of tracheal bifurcation to below the base of the heart using prospective ECG triggering with the centre of the acquisition at 70% of the R-R interval. From the raw data, the images were reconstructed with standard kernel in 3 mm thick axial, non-overlapping slices and 25 cm field of view. All image analyses were performed on a dedicated workstation (Aquarius 3D Workstation, TeraRecon, San Mateo, CA, USA). A coronary calcified lesion was defined as an area with a density >130 HU and covering at least 6 pixels. The Agatston method was used to determine the coronary calcium score (CCS) by multiplying each lesion area by a weighted CT attenuation score in the lesion.

### Quantification of pericardial (PCF) and thoracic peri-aortic adipose tissue volume (TAT)

Pericardial (PCF) and thoracic peri-aortic adipose tissue (TAT) volumes were quantified from the heart CT scan using a dedicated workstation (Aquarius 3D Workstation, TeraRecon, San Mateo, CA, USA). The semi-automatic segmentation technique was developed for quantification of adipose tissue volumes. We traced the region of interest manually and defined adipose tissue as pixels within a window of -195 HU to −45 HU and a window centre of −120 HU. PCF was defined as any adipose tissue located within the pericardial sac. TAT tissue was defined as all of the adipose tissue surrounding the thoracic aorta extending 67.5 mm caudally from the level of the bifurcation of pulmonary arteries. This approach has previously been validated.[5]–[7] The intra-observer and inter-observer coefficient of variation were 4.27%, 4.87% and 6.58%, 6.81% for PCF and TAT, respectively. Both observers performed an independent reading in a random subset of 40 subjects[5].

### Body Fat Composition Analysis

Body fat composition analysis in our study was assessed by utilizing the bioelectrical impedance from foot-to-foot measure using a Tanita-305 body fat analyzer (Tanita Corporation, Tokyo, Japan). Output variables were total body weight, impedance, fat mass, fat-free mass, and body fat composition as calculated by the manufacturer's software.

### Statistical Analysis

Continuous data were expressed as the mean and standard deviation with categorical data expressed as the frequency and proportion of occurrence. Differences of baseline demographics between groups were tested by Student t- test, with categorical data analyzed by chi-square or Fisher's exact test as appropriate. The Wilcoxon non-parametric trend test was used to estimate the trend of all continuous data and ordinal variables across all ordered groups. One-way ANOVA was performed to test the differences in the continuous data among groups with post-hoc Bonferroni correction analysis performed for paired comparisons.

Univariable logistic regression was used to examine the odds of having various metabolic abnormalities according to tertile of visceral adipose tissue volume or body fat composition, with the lowest tertile as the referent group. Univariable linear regression was used to examine the relationship of body fat composition and measures of visceral adiposity with various clinical variables, anthropometric measures, and serum biomarkers of metabolism. Several multivariable linear regression models were then constructed to examine the independent relationships of body fat composition and measures of visceral adiposity with hs-CRP or coronary calcium score separately after adjustment for clinical variables, anthropometric measures and lifestyle risk factors.

The diagnostic utility for measures of adiposity for predicting metabolic syndrome was assessed using receiver operating characteristic (ROC) curves with optimal cut-offs chosen by the maximum summation of individual sensitivity and specificity. We further compared the area under the ROC curve (AUROC) for each measure of adiposity.

Data were analyzed using STATA 8.2 (STATA Corp., College Station, Texas). All statistical tests were two-sided with a 0.05 significance level.

## Results

### Baseline characteristics of study participants

**Figure 1:**
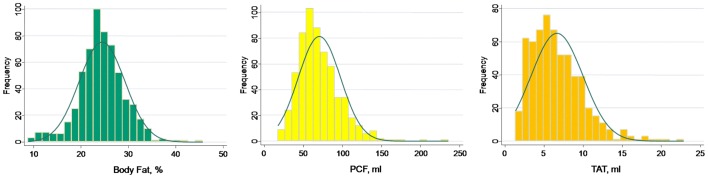
Distributions of body fat composition and both visceral adipose tissue volumes (PCF and TAT) in our study cohort. PCF, pericardial adipose tissue; TAT, thoracic peri-aortic adipose tissue.

**Figure 2:**
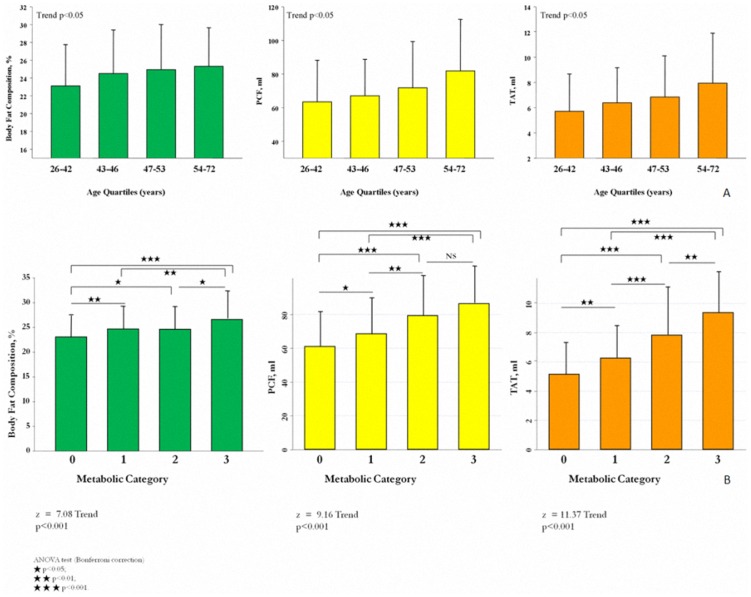
Comparison of body fat composition, pericardial and thoracic peri-aortic adipose tissue with age quartiles and numbers of abnormal metabolic components. A ) Increases in adiposity across age quartiles in our cohort (all p for trend <0.05). **B**). Larger numbers of abnormal metabolic components were associated with higher body fat composition and increasing visceral adipose tissue burden (all p for trend <0.001). Metabolic category 0, metabolic score = 0; 1, metabolic score = 1; 2, metabolic score = 2; 3, metabolic score ≥3. Abbreviations as in Figure 1. ★p<0.05, ★★p<0.01, ★★★p<0.001 by ANOVA post hoc paired comparison

A total of 608 subjects met our entry criteria and were enrolled in this study. The mean age was 47.3±7.1 years with 164 (27%) female. Baseline demographic data across increasing age quartiles were displayed in table 1. In our cohort, 130 (21.4%) subjects had CCS of zero, with 478 (78.6%) had CCS above zero (mean value: 22.1, 25^th^ & 75^th^: 2.3 & 32.4, respectively). Increasing age was associated with higher systolic and diastolic blood pressure, widened pulse pressure, decreased height, greater waist circumference, higher fasting glucose, higher HbA1c, higher total cholesterol, higher low-density lipoprotein, reduced eGFR, and decreased prevalence of alcohol use (all trend p<0.05). The distributions of body fat composition, PCF and TAT were shown in Figure 1, with mean body fat composition, PCF, and TAT estimated to be 24.4±4.8%, 71.85±27.4 ml, and 6.9±3.5 ml, respectively. Increasing amounts of each adiposity measure were observed with increasing age (Figure 2A, all trend p<0.05). For each decade increase in age, there were 8.07 ml and 13.55 ml increases in PCF and 1.53 ml and 1.19 ml increases in TAT for men and women, respectively.

**Table 1:** Baseline characteristics of the study population by age quartiles.

Age Quartiles	Quartile 1	Quartile 2	Quartile 3	Quartile 4	
Age, years	26 to 42	43 to 46	47 to 53	54 to 72	Trend p
***Baseline Information***					
Gender (female), %	44 (26.5%)	28 (20%)	52 (30.1%)	40 (31%)	0.146
SBP, mmHg	114.2±12.5	114.9±14.2	118.7±15	122.7±15.4	<0.001
DBP, mmHg	72.9±9.6	74±10.2	75.5±9.8	76.3±9.2	<0.001
Pulse Pressure, mmHg	0.87±0.07	0.88±0.07	0.89±0.06	0.89±0.07	0.005
Pulse Rate, 1/min	72.6±7.7	73.6±9.4	72.2±8	72.9±9	0.69
***Anthropometric Measures***					
Body Height, cm	167.4±7.3	168.3±7.4	165.4±7.8	164.6±8.7	<0.001
Body Weight, kgw	66.5±12.4	68.1±9.9	66.2±10.8	65.7±11.4	0.31
BMI, kg/m^2^	23.6±3.3	24±2.7	24.1±2.9	24.1±2.8	0.08
Waist circumference, cm	80.4±10.2	82.6±8.6	83±8.5	83.5±8.9	0.02
BSA, m^2^	1.67±0.25	1.73±0.21	1.66±0.23	1.65±0.23	0.17
***Biochemical Data and Cardiometabolic Markers***					
Fasting glucose, mg/dL	93.3±10.5	94.4±9.0	98.9±22.1	98.1±11	<0.001
HbA1c, %	5.62±0.33	5.71±0.34	5.78±0.54	5.88±0.43	<0.001
Cholesterol, mg/dL	190.3±33.5	189.7±34	203.2±37.7	200.5±32.6	<0.001
TG, mg/dL	131.9±87.4	138.3±74.2	139.3±104.7	131.6±69.8	0.52
LDL, mg/dL	121.5±31	125.1±32	133.7±33	132.4±27.4	<0.001
HDL, mg/dL	52.6±13.6	50±14	53.2±13.8	52.1±12.5	0.62
Insulin, mIU/mL	5.4±2.8	5.2±3.0	5.5±2.4	5.3±2.5	0.69
HOMA-IR, IU/mL	1.27±0.63	1.28±1.0	1.3±0.74	1.3±0.8	0.82
eGFR, ml.min. 73 m^2^	93.7±16.8	90±15.8	90.2±15.8	85.2±15.8	<0.001
***Lifestyle risk factors***					
Smoking, %	39 (23.5%)	36 (25.7%)	38 (22%)	28 (21.7%)	0.846
Exercise, %	42 (25.3%)	37 (26.4%)	48 (27.8%)	28 (21.7%)	0.681
Alcohol use, %	23 (13.9%)	24 (17.1%)	51 (29.5%)	19 (14.7%)	0.001

BMI: body mass index; BSA: body surface area; DBP: diastolic blood pressure; eGFR: estimated glomerular filtration rate; HbA1c: glycosylated hemoglobin level; HDL: high-density lipoprotein; HOMA-IR: homeostasis model assessment-estimated insulin resistance; LDL: low-density lipoprotein; SBP: systolic blood pressure; TG: Triglycerides.

### The association between visceral adipose tissue volume, anthropometrics, biochemical data and systemic inflammation

In addition to age, both PCF and TAT volumes were also positively correlated with blood pressure, blood glucose, HbA1C, triglycerides, LDL, uric acid, homocysteine (Table 2, all p<0.05) and were inversely associated with HDL and eGFR (both p<0.05). In general, anthropometric measures were more strongly correlated with PCF and TAT than percent body fat. There were modest positive correlations between PCF, TAT and serum markers of inflammation (hs-CRP and WBC count, p<0.05).

**Table 2:** Relationship between clinical variables, anthropometric measures and measures of various adiposity.

	ß-coef. (1 SD increase)	ß-coef. (1 SD increase)	ß-coef. (1 SD increase)
Variables	Body Fat Composition, %	PCF, ml	TAT, ml
Age, years	0.16*	0.25*	0.27*
SBP, mmHg	0.06	0.25*	0.33*
DBP, mmHg	0.01	0.20*	0.32*
BMI, kg/m^2^	0.34*	0.44*	0.55*
Weight, kg	0.14*	0.46*	0.58*
Waist, cm	0.23*	0.49*	0.64*
BSA, m^2^	0.19*	0.43*	0.54*
Fasting Glucose	0.04	0.16*	0.27*
Post-Prandial Glucose	0.06	0.12*	0.19*
HbA1C, %	0.11*	0.14*	0.22*
Insulin, mIU/mL	−0.07	0.11^¥^	0.17*
HOMA-IR, IU/mL	−0.08	0.14*	0.22*
Cholesterol, mg/dL	0.18*	0.15*	0.08^¥^
TG, mg/dL	0.14*	0.17*	0.30*
HDL, mg/dL	−0.11*	−0.25*	−0.41*
LDL, mg/dL	0.2*	0.19*	0.15*
Uric Acid, mg/dL	−0.03	0.21*	0.38*
Homocysteine, mg/dL	−0.14^¥^	0.20*	0.25*
eGFR	−0.001	−0.14*	−0.24*
Hs-CRP	0.01	0.17*	0.24*
WBC Count	0.07^¥^	0.13*	0.24*

hs-CRP, high sensitivity C-reactive protein; WBC Count, circulating white blood cell count; PCF, pericardial adipose tissue; TAT, thoracic peri-aortic adipose tissue. Other abbreviations as in Table 1.

* p<0.05, ^¥^p<0.1.

### The association between body fat composition, visceral adipose tissue volume and individual abnormal metabolic components

**Figure 3:**
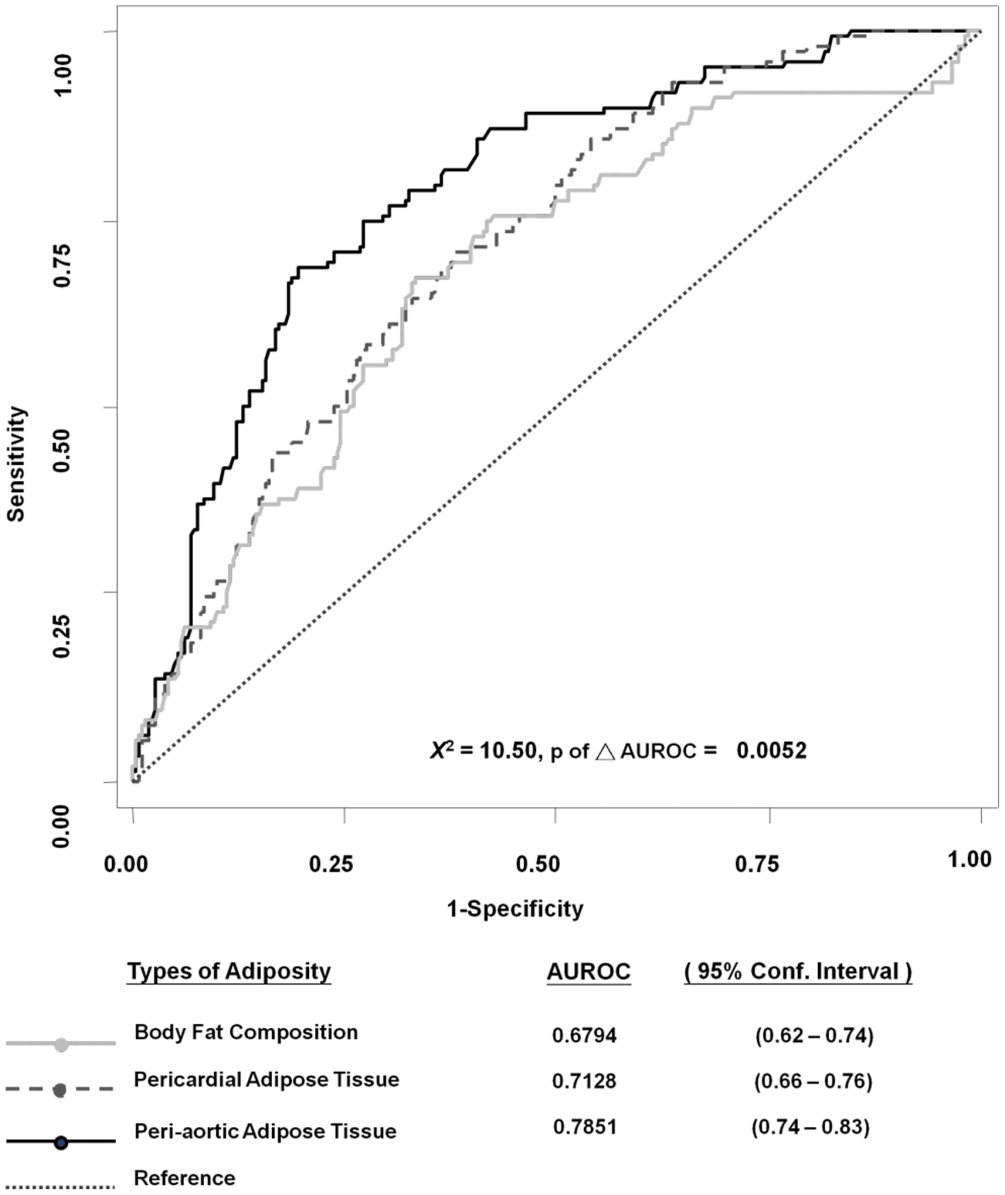
Receiver operating characteristic (ROC) curves for each measure of adiposity in identifying metabolic syndrome, with individual 95% confidence interval (95% CI) reported . Furthermore, the difference between each area under ROC (AUROC) was calculated with p value provided for statistical significance (p of ΔAUROC). The area under the ROC curve (AUROC) was largest for peri-aortic adipose tissue (p of ΔAUROC: 0.00562).

Ninety-nine subjects (16.3%) met criteria for MetS. Table 3 displays the risks of individual abnormal metabolic components for MetS according to tertiles of each measure of adiposity. Subjects in the highest tertiles of PCF and TAT were more likely to have abnormal MetS components compared to the lowest tertiles (all p<0.05). In addition, higher metabolic scores were associated with higher percent body fat composition, PCF, and TAT volumes (Figure 2B, all p for trend <0.05). Compared to those without MetS (metabolic scores < = 2), those with MetS (metabolic scores ≥3) had significantly larger body fat composition and TAT volume (both ANOVA p<0.05), though no significant difference was observed in PCF volume (p = NS). The optimal cut-offs for identifying MetS by adipose tissue were 8.29 ml for TAT (sensitivity: 68.7%, specificity: 80.5%), 71.67 ml for PCF (sensitivity: 68.7%, specificity: 63.8%), and 24.56% for body fat composition (sensitivity: 74.5%, specificity: 58.2%). Compared to body fat composition and PCF, the area under the ROC curve to predict metabolic syndrome in our cohort was highest for TAT (Figure 3).

**Table 3:** Odds of having abnormal metabolic markers according to tertiles of adiposity measures.

	Abnormal Waist		Abnormal TG		Abnormal HDL		Abnormal BP		Abnormal Glucose		
Tertiles (Strata)	OR	95% CI	OR	95% CI	OR	95% CI	OR	95% CI	OR	95% CI	
**Body Fat Composition (%)**											
8.8–22.7, median: 20.7	1	-	1	-	1	-	1	-	1	-	
22.7–26.2, median: 24.3	1.8	1.1–3.1*	1.9	1.2–3.1*	2.7	1.2–6.0	1.1	0.7–1.7	2.3	1.4–3.7*	
26.2–45.7, median: 28.5	6.1	3.7–10*	3.3	2.1–5.1*	6.5	3.1–13.6*	0.9	0.6–1.5	1.8	1.1–2.9*	
**PCF (ml)**											
17.4–57.2, median: 46.7	1	-	1	-	1	-	1	-	1	-	
57.3–78.2, median: 65.8	3.1	1.8–5.3*	2.7	1.8–4.1*	1.3	0.8–2.1	1	0.5–1.8	2.2	1.4–3.4*	
78.3–235.7, median: 96.1	7.2	4.3–12.1*	2.9	1.8–4.6*	1.6	1.0–2.7*	2.1	1.2–3.7*	3.2	2.0–5.0*	
**TAT (ml)**											
1.4–4.9, median: 3.3	1	-	1	-	1	-	1	-	1		-
4.9–7.7, median: 6.2	1.8	1.1–2.9*	2.1	1.4–3.3*	1.4	0.9–2.3	1.9	1.0–3.6*	2		1.2–3.3*
7.7–22.9, median: 9.6	4.7	2.9–7.5*	5.3	3.4–8.3*	2	1.3–3.2*	3.7	2.0–6.9*	4.1		2.5–6.7*

* p<0.05, ^¥^p<0.1.

OR, odds ratio; CI, confidence interval. Other abbreviations as in Tables 1 and 2.

### The relationship between visceral adiposity tissue volume and circulating white blood cell (WBC) counts, serum insulin, hs-CRP level and coronary calcium score (CCS)

**Figure 4:**
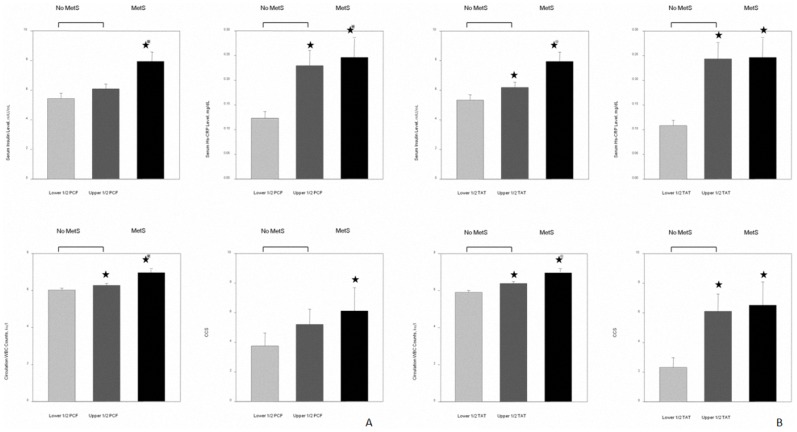
Comparison of serum insulin level, high sensitivity C-reactive protein (hs-CRP), circulating white blood cell (WBC) count and coronary calcium score (CCS) among subjects with and without metabolic syndrome. Those without metabolic syndrome are further divided into upper and lower halves of visceral adipose tissue volume. **A**) Pericardial adipose tissue (PCF); mean volumes in the three groups were 48.3±10.2, 86.9±23.3 and 86.4±25.6 ml, respectively. **B**) Thoracic peri-aortic adipose tissue (TAT); mean volumes in the three groups were 3.8±1.1, 8.4±2.7 and 9.4±3.3 ml, respectively. ★p<0.05 vs lower visceral adipose tissue volume without metabolic syndrome, ★p<0.05 vs upper visceral adipose tissue volume without metabolic syndrome (Kruskal-Wallis test with post hoc paired comparison).

**Figure 5:**
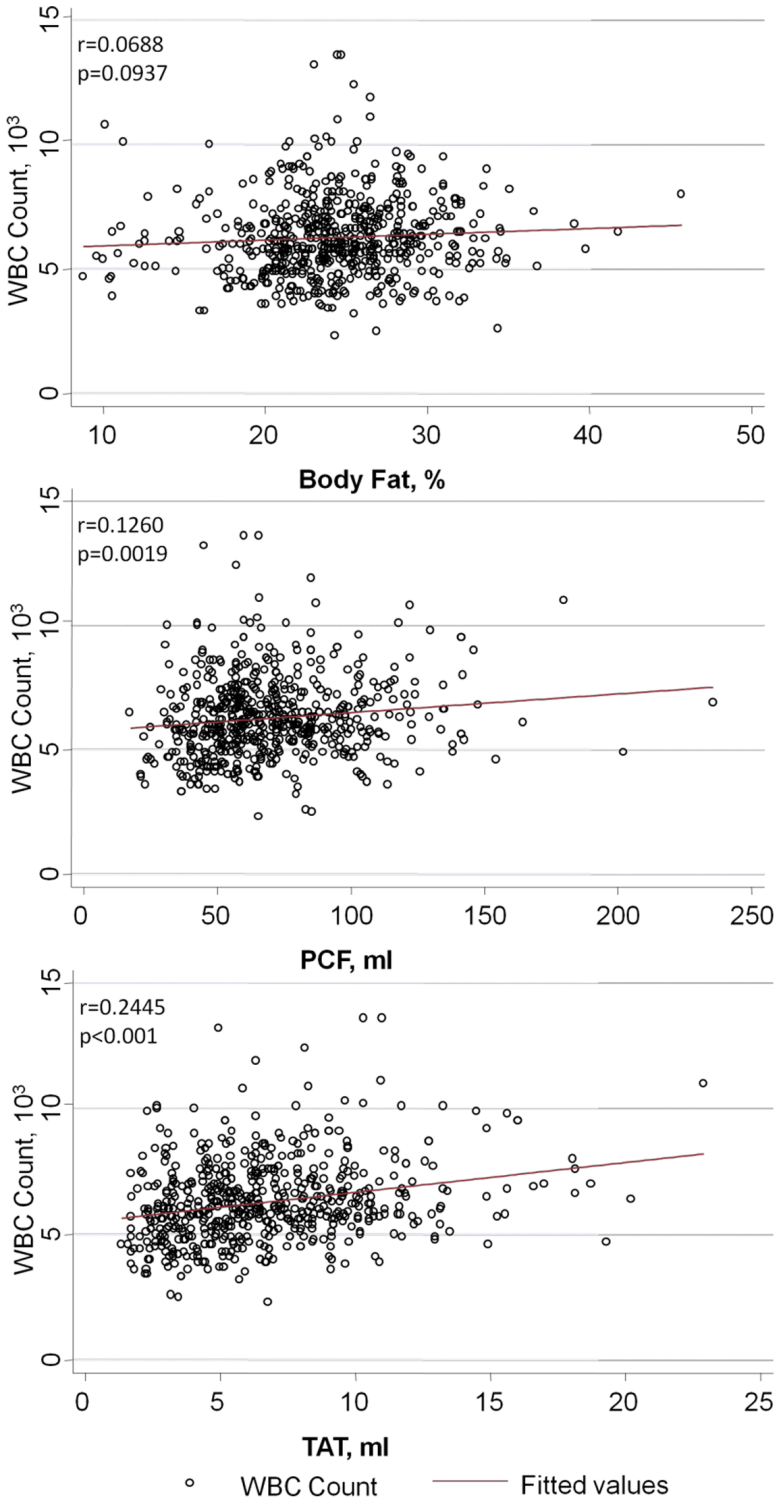
Correlation between measures of various adiposity (body fat composition and both visceral adipose tissue volume) and circulating WBC counts. Though body fat composition and visceral adipose tissue volumes had positive linear correlations with WBC numbers, only the correlation with TAT remained significant after multivariable adjustment. Clinical variables in the model included age, gender, systolic blood pressure, fasting glucose, cholesterol, high-density lipoprotein, estimated glomerular filtration rate, exercise, smoking and alcohol consumption. BMI, body-mass index; BSA; body surface area; WBC, white blood cell; PCF, pericardial adipose tissue; TAT, thoracic peri-aortic adipose tissue.

Figures 4A & 4B demonstrated the differences of serum insulin level, systemic inflammation in terms of hs-CRP, circulating WBC counts and CCS in our study subjects with and without MetS. For those without MetS, subjects were further categorized into upper and lower half of visceral adiposity volume. The 3D-based volume measure of visceral adipose tissue among these three groups were 48.3±10.2, 86.9±23.3 & 86.4±25.6 ml for PCF and 3.8±1.1, 8.4±2.7 & 9.4±3.3 ml for TAT, respectively. In brief, all participants who had MetS had generally higher serum insulin, hs-CRP and circulating WBC as well as higher CCS (all p<0.05). However, for those without MetS, subjects categorized into the upper half of visceral adipose tissue volume (either PCF or TAT) tended to have higher levels of each of these measures. These relationships were more prominent for TAT (Figure 4B, all p<0.05), in comparison to PCF (Figure 4A, p<0.05 for hs-CRP and WBC only). Both visceral adipose tissue volume but not body fat composition had a positive linear relationship with total leukocyte counts (Figure 5); however, only TAT demonstrated consistent and independent association after adjustment for clinical variables and anthropometric measurements including BMI, waist circumference and BSA (all p<0.05).

In uni-variable linear regression models, both PCF and TAT were associated with hs-CRP though only TAT showed correlation with CCS (Table 4). In multivariable models, the relationship of PCF with hs-CRP was attenuated (all p>0.05). However, the relationship of TAT with both hs-CRP and CCS remained significant even after adjustment for age, gender, various anthropometric measures, traditional clinical variables and lifestyle risk factors (all p<0.05). Instead, there was no such independent association between PCF and coronary calcium scores in all multivariable models except for a borderline inverse association after modeling for age, gender, blood pressure, BMI and waist circumference (p> = 0.05 & <0.1).

**Table 4:** The association of adiposity measures with hs-CRP and coronary calcium score after adjustment for age, gender, and anthropometric data.

	Serum hs-CRP Level		
Unadjusted Model	Body Fat Composition, %	PCF, ml	TAT, ml
ß-coef. (1 SD increase)	0.004	0.19*	0.26*
**Multivariate Model (Age & Gender adjusted)**			
1. ß-coef. (1 SD increase)	0.06	0.16*	0.26*
**Multivariate Model (Modeling for BMI)**			
2. ß-coef. (1 SD increase)	−0.004	0.05	0.14*
3. ß-coef. (1 SD increase)	0.01	0.06	0.14*
**Multivariate Model (Modeling for BSA)**			
2. ß-coef. (1 SD increase)	0.14^¥^	0.13^¥^	0.20*
3. ß-coef. (1 SD increase)	0.17^¥^	0.11^¥^	0.21*
**Multivariate Model (Modeling for Waist)**			
2. ß-coef. (1 SD increase)	0.002	0.07	0.15*
3. ß-coef. (1 SD increase)	0.01	0.09	0.19*

	Coronary Calcium Score (CCS)		
Unadjusted Model	Body Fat Composition, %	PCF, ml	TAT, ml
ß-coef. (1 SD increase)	0.07^¥^	0.06	0.23*
**Multivariate Model (Age & Gender adjusted)**			
1. ß-coef. (1 SD increase)	0.08^¥^	−0.04	0.13*
**Multivariate Model (Modeling for BMI)**			
2. ß-coef. (1 SD increase)	0.06	−0.08^¥^	0.13*
3. ß-coef. (1 SD increase)	0.09^¥^	−0.07	0.18*
**Multivariate Model (Modeling for BSA)**			
2. ß-coef. (1 SD increase)	0.09*	−0.05	0.13*
3. ß-coef. (1 SD increase)	0.13*	−0.04	0.18*
**Multivariate Model (Modeling for Waist)**			
2. ß-coef. (1 SD increase)	0.07	−0.08^¥^	0.14*
3. ß-coef. (1 SD increase)	0.09^¥^	−0.07	0.19*

Abbreviations as in Table 1 & 2. SD, standard deviation.

Model 1; Adjusted for age & gender;

Model 2: Adjusted for age, gender, systolic blood pressure and anthropometrics (BMI, BSA & Waist, sequentially);

Model 3: Adjusted for age, gender, systolic blood pressure, cholesterol, fasting glucose, eGFR, HDL, exercise, drinking, smoking behavior & anthrpometrics (BMI, BSA & Waist, sequentially).

* denotes p<0.05, ^¥^denotes p> = 0.05 & <0.1.

## Discussion

This cross-sectional study comprehensively describes the clinical significance of age-related changes in body fat composition and visceral adipose tissue accumulation in a relatively healthy population. We demonstrate a direct and positive linear relationship between increasing age and both visceral adipose tissue volumes, which are linked to several metabolic derangements and systemic inflammation. Moreover, TAT, but not PCF, remained independently associated with markers of systemic inflammation and subclinical coronary atherosclerosis even after adjustment for traditional risk factors and anthropometric measures.

Aging is tightly linked to increased abdominal fat accumulation in both men and women[12], [18]. This shift of adipose tissue to visceral and other ectopic sites[9], [10], [18] may partly explain the higher prevalence of metabolic syndrome in the elderly population[8]. In addition, waist circumference is strongly associated with hs-CRP and cardiovascular morbidity in the elderly[14], [19], [23]–[25]. Prior studies suggest that hs-CRP is approximately 2-fold higher in subjects older than 65 years compared to those in middle-aged groups.[15] Central obesity caused by excessive abdominal visceral adipose tissue burden is a powerful independent risk factor for the development of cardiovascular disease.[26] Currently, the most common method of assessing abdominal adiposity in clinical practice is to measure waist circumference, though this method is limited by the inability to differentiate subcutaneous from visceral adipose tissue when compared to CT-derived methods.[12], [27], [28]

Whether the pro-inflammatory activity of visceral adipose tissue or other ectopic adipose tissues is related to the total volume, or “mass effect”, remains unclear. [23] Abdominal VAT is typically quantified by measuring the thickness or area of adipose tissue from a single slice of an abdominal CT, though this method does not necessarily measure the true total amount of VAT. [9], [12], [29], [30] In this regard, we sought to test whether three-dimensional, volumetric measurements of two commonly assessed visceral fat depots (PCF and TAT) could differentiate more pathologic adiposity in an apparently healthy population. In fact, we observed that only TAT volume was independently associated with markers of systemic inflammation and subclinical atherosclerosis. On the other hand, PCF did not appear to be associated with subclinical atherosclerosis or serum markers of systemic inflammation in fully-adjusted models. This is not altogether unexpected, as the association between pericardial adipose tissue and coronary atherosclerosis has been inconsistent in prior studies.[31], [32] This may be explained by the potential “dual role” (both harmful and protective) of cardiovascular risks from pericardial adipose tissue in certain populations or by the methods used for adipose tissue quantification. In our cohort, participants were relatively healthy, non-obese, and free from history or symptoms of heart disease. Our data are consistent with a model where the quality and quantity of pericardial fat is in balance during health, but becomes unbalanced in pathologic disease states. Finally, our 3D quantification method may result in the inclusion of qualitatively different regions of visceral fat with potentially varied biological effects or metabolic activity which may represent a more global measure of this heterogenous adipose tissue depot.[33]

The age-related accumulation of TAT and its independent relationship with circulating markers of inflammation may be explained by its spatial distribution and anatomic adjacency to large arteries. Expansion of peri-vascular adipose tissue and its infiltration by macrophages and T lymphocytes has been demonstrated in subjects with obesity and atherosclerosis.[34], [35] Not being confined in the pericardial sac, inflammatory cytokines produced in peri-aortic adipose tissue may more easily diffuse through the adventitia layer across the arterial wall from outside-to-inside.[33], [36], [37] In this manner, inflammatory cytokines may be directly released into the circulation where subsequent endothelial inflammatory activation and dysfunction could occur in distant vascular beds.[32] In this study, we have demonstrated that in subjects without frank metabolic syndrome, higher TAT volume was able to differentiate subjects with higher levels of several metabolic risk markers. TAT volume may therefore serve as an early marker of cardiometabolic risk in apparently healthy individuals prior to the development of overt cardiovascular disease.

There are several limitations of our study. Our cohort included fewer women than men, which may limit its generalizability. In addition, as in every cross-sectional study, we cannot prove that the observed relationships between site-specific visceral adiposity and cardiometabolic risk are causal. Future longitudinal studies or interventions to reduce visceral adipose tissue burden in this population may help to clarify these relationships.

## Conclusion

Age-related changes in adiposity, including increases in body fat composition and visceral adipose tissue volumes, are associated with cardiometabolic risk profiles prior to the development of overt cardiovascular diseases. More importantly, our findings suggest that such visceral adipose tissue accumulation may have distinct biological effects depending on its location. Compared to pericardial adipose tissue and other measures of adiposity, peri-aortic adipose tissue is more closely associated with cardiometabolic risk and subclinical atherosclerosis in a relatively healthy population.

## Supporting Information

Materials S1 **Supplemental Material.** This includes: Supplementary Materials. Figure S1: A) Identification of the pericardium (white arrow) and additional landmarks, including right coronary artery (dot arrow) in the axial view. B) Traced pericardium from the level of left main coronary artery to the level of cardiac base (gray scale area). C) Three dimensional (3D) reconstruction of a 6 mm thick axial CT slice. D) Whole volume quantification of all axial slices from the left main coronary artery to the cardiac base. Figure S2: A) Traced peri-aortic adipose tissue surrounding the thoracic aorta in the axial view. B) Region of interest from the cross-sectional plane (gray scale area). C) Three dimensional (3D) reconstruction of a 6 mm thick CT slice. D) Whole volume quantification of slices extending 67.5 mm caudally from the bifurcation of the pulmonary artery.
(PDF)
