# Supplementary material for: The Normal Limits, Subclinical Significance, Related Metabolic Derangements and Distinct Biological Effects of Body Site-Specific Adiposity in Relatively Healthy Population
Source: PLoS One. 2013 Apr 19;8(4):e61997. doi: 10.1371/journal.pone.0061997 (PMC3631150; doi:10.1371/journal.pone.0061997)

Supplemental Material

The three-dimensional volume quantification of pericardial and thoracic peri-aortic adipose tissue (PCF and TAT) was performed on a dedicated offline workstation (Aquarius 3D TeraRecon, San Mateo, CA, USA). We traced the region of interest manually using anatomic landmarks of the heart and thoracic descending aorta as described below. A threshold-based algorithm was applied to identify pixels containing adipose tissue (defined as Hounsfield Units within the range of -195 to -45), and the adipose tissue volume was then quantified as the volume of these pixels contained within the defined region of interest.

Supplemental Figure 1:

A) Identification of the pericardium (white arrow) and additional landmarks, including right coronary artery (dot arrow) in the axial view. B) Traced pericardium from the level of left main coronary artery to the level of cardiac base (gray scale area). C) Three dimensional (3D) reconstruction of a 6mm thick axial CT slice. D) Whole volume quantification of all axial slices from the left main coronary artery to the cardiac base.

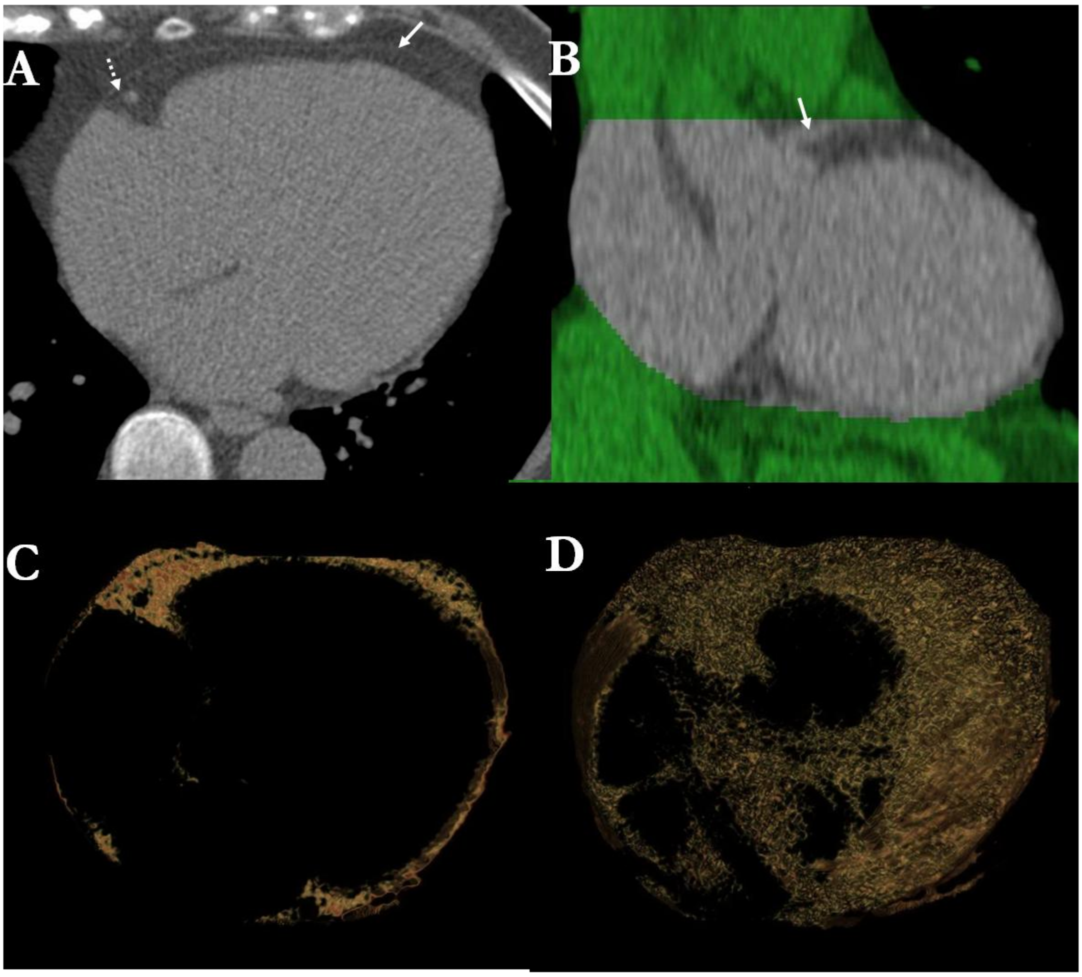

Supplemental Figure 2:

A) Traced peri-aortic adipose tissue surrounding the thoracic aorta in the axial view. B) Region of interest from the cross-sectional plane (gray scale area). C) Three dimensional (3D) reconstruction of a 6mm thick CT slice. D) Whole volume quantification of slices extending 67.5mm caudally from the bifurcation of the pulmonary artery.

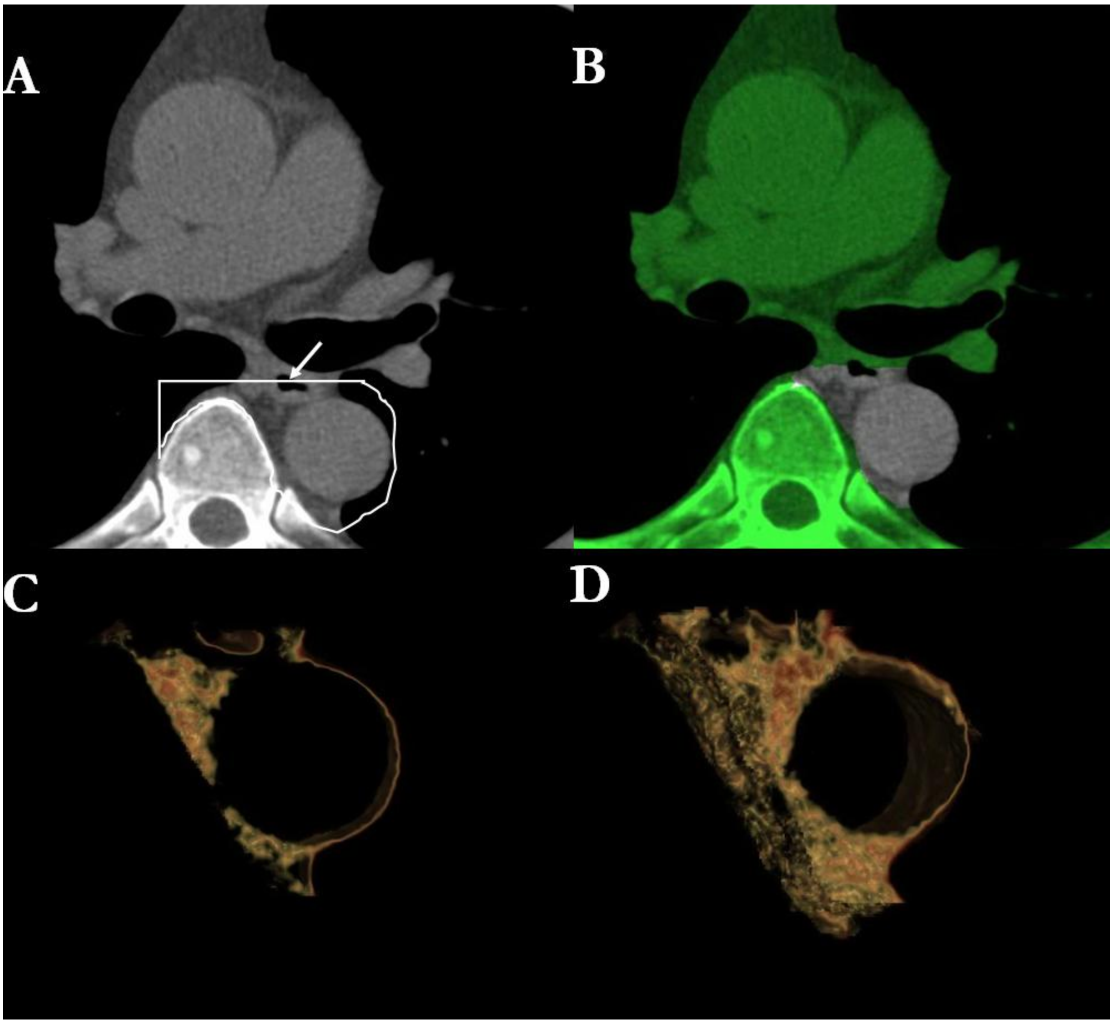

Supplement: Materials S1 — Supplemental Material. This includes: Supplementary Materials. Figure S1: A) Identification of the pericardium (white arrow) and additional landmarks, including right coronary artery (dot arrow) in the axial view. B) Traced pericardium from the level of left main coronary artery to the level of cardiac base (gray scale area). C) Three dimensional (3D) reconstruction of a 6 mm thick axial CT slice. D) Whole volume quantification of all axial slices from the left main coronary artery to the cardiac base. Figure S2: A) Traced peri-aortic adipose tissue surrounding the thoracic aorta in the axial view. B) Region of interest from the cross-sectional plane (gray scale area). C) Three dimensional (3D) reconstruction of a 6 mm thick CT slice. D) Whole volume quantification of slices extending 67.5 mm caudally from the bifurcation of the pulmonary artery. (PDF) [file pone.0061997.s001.pdf]
